# Suppressing the Photocatalytic Activity of TiO_2_ Nanoparticles by Extremely Thin Al_2_O_3_ Films Grown by Gas-Phase Deposition at Ambient Conditions

**DOI:** 10.3390/nano8020061

**Published:** 2018-01-24

**Authors:** Jing Guo, Hao Van Bui, David Valdesueiro, Shaojun Yuan, Bin Liang, J. Ruud van Ommen

**Affiliations:** 1 Product & Process Engineering, Department of Chemical Engineering, Faculty of Applied Sciences, Delft University of Technology, 2629 HZ Delft, The Netherlands; guojing0519@hotmail.com (J.G.); d.valdesueiro@delft-imp.nl (D.V.); J.R.vanOmmen@tudelft.nl (J.R.v.O.); 2 Multi-Phase Mass Transfer & Reaction Engineering Lab, College of Chemical Engineering, Sichuan University, Chengdu 610065, China; ysj@scu.edu.cn (S.Y.); liangbin@scu.edu.cn (B.L.); 3 Delft IMP B.V., Molengraaffsingel 10, 2629 JD Delft, The Netherlands; 4 Shanxi Province Key Laboratory of Higee-Oriented Chemical Engineering, North University of China, Taiyuan 030051, China; 5 Department of Physics, Quy Nhon University, 170 An Duong Vuong Street, Quy Nhon City 590000, Vietnam

**Keywords:** ultrathin Al_2_O_3_ films, atomic layer deposition, fluidized bed reactor, photocatalytic suppression, TiO_2_ pigments

## Abstract

This work investigated the suppression of photocatalytic activity of titanium dioxide (TiO_2_) pigment powders by extremely thin aluminum oxide (Al_2_O_3_) films deposited via an atomic-layer-deposition-type process using trimethylaluminum (TMA) and H_2_O as precursors. The deposition was performed on multiple grams of TiO_2_ powder at room temperature and atmospheric pressure in a fluidized bed reactor, resulting in the growth of uniform and conformal Al_2_O_3_ films with thickness control at sub-nanometer level. The as-deposited Al_2_O_3_ films exhibited excellent photocatalytic suppression ability. Accordingly, an Al_2_O_3_ layer with a thickness of 1 nm could efficiently suppress the photocatalytic activities of rutile, anatase, and P25 TiO_2_ nanoparticles without affecting their bulk optical properties. In addition, the influence of high-temperature annealing on the properties of the Al_2_O_3_ layers was investigated, revealing the possibility of achieving porous Al_2_O_3_ layers. Our approach demonstrated a fast, efficient, and simple route to coating Al_2_O_3_ films on TiO_2_ pigment powders at the multigram scale, and showed great potential for large-scale production development.

## 1. Introduction

The brilliant white color and high photostability of nanoparticulate titanium dioxide (TiO_2_) make it an excellent white pigment in the paint, plastic, and paper industries [1]. In particular, TiO_2_ has been widely used as a white pigment in oil paint since the 20th century, replacing lead white—the most important white pigment in history [2]. However, the high photocatalytic activity of TiO_2_ under UV light irradiation causes the inevitable degradation of surrounding materials, which consequently changes the appearance and severely decreases the lifetime of the paintings. Depending on the surrounding materials, the photocatalytic degradation can occur via different regimes [3]. For instance, photocatalytic reactions can either promote polymerization of the organic binder, creating cross-linking that leads to embrittlement [4], or decompose the binder into volatile organic components, resulting in surface roughening and chalking [5]. In addition, photocatalytic reactions can induce degradation of other organic colored pigments, which consequently leads to discoloration [6]. Photocatalytic degradation has also been observed for plastic art objects and photographic papers using TiO_2_ as a white pigment [7]. Therefore, to avoid the photodegradation caused by the high catalytic activity of TiO_2_, in these applications TiO_2_ particles are commonly coated with a thin layer of a ceramic oxide, such as Al_2_O_3_ and SiO_2_, with a thickness of a few nanometers [8,9,10,11,12,13,14,15,16]. On the one hand, owing to their good insulating properties, ultrathin layers of these oxides can provide efficient photocatalytic suppression by preventing the transport of electrons and holes generated by UV-light absorption to the surface. On the other hand, the large bandgap of the ceramic oxide materials ensures the optical transparency of the coating layers, and conserves the brilliant white color of TiO_2_.

Ceramic oxide coating on TiO_2_ pigments to mitigate their photocatalytic activity began in the early 1940s, using a precipitation technique [17]. Since then, a number of methods have been developed for the deposition of ultrathin coating layers. Wet chemistry methods, such as sol-gel and precipitation, have been most popular due to their simplicity, inexpensiveness, and versatility in depositing thin films of various materials. For example, thin films of single SiO_2_ layer or binary Al_2_O_3_/SiO_2_ layers can be obtained by precipitation [11], whereas sol-gel enables the deposition of various ceramic and transition metal oxides such as SiO_2_, Al_2_O_3_, ZrO_2_, NiO, and CoO with tunable morphology and properties [9,10,18,19]. However, wet chemistry methods have several shortcomings in controlling the coating thickness and conformity due to their high sensitivity to experimental parameters, such as precursor concentration, type and pH of the solvents, deposition time, and temperature. In addition, wet chemistry methods are time-consuming and commonly require post-treatment processes, for instance, high-temperature treatment, washing, drying, and separation to eliminate impurities arising from the residual solvent and reaction byproducts [9,10,14]. Moreover, when it comes to large-scale production, these techniques usually encounter environmental issues due to the use of a large amount of solvents and chemical solutions. Therefore, there has been a constant search for a simple, fast, environmentally friendly, and controllable deposition method that can overcome the drawbacks associated with wet chemistry methods, while being feasible for large-scale production.

Atomic layer deposition (ALD) is a gas-phase deposition technique that is carried out using alternating exposures of the substrate to chemical reactants, each having self-limiting surface reactions that enable the control of film thickness at the atomic level [20,21,22]. ALD has been developed for a few decades [23,24], and utilized in the deposition of a wealth of materials on virtually every substrate for applications in various fields such as microelectronics, catalysis, and energy conversion and storage [21,25,26]. Particularly for coating on nanoparticles, ALD has emerged as an excellent method for the deposition of ultrathin conformal films of SiO_2_ and Al_2_O_3_ [14,15,24,27,28,29,30,31,32,33,34,35,36]. In particular, recent developments in ALD reactor types, such as rotary and fluidized bed reactors (FBR), have enabled ALD of thin films on large quantities of micro- and nanoparticles [37,38,39]. Particularly, ALD of SiO_2_ and Al_2_O_3_ on TiO_2_ nanoparticles to mitigate their photocatalytic activities for pigment applications has been investigated by Weimer’s research group [14,15,40]. Using tris-dimethylaminosilane (TMAS) and H_2_O_2_ as precursors, King et al. demonstrated that ultrathin conformal SiO_2_ films could be deposited onto anatase/rutile TiO_2_ powders with a growth-per-cycle (GPC) of approximately 0.04 nm at 500 °C [14]. Such an ALD-grown SiO_2_ film with a thickness of 2 nm could suppress 98% of the photocatalytic activity of anatase TiO_2_. However, the low GPC is not favorable for large-scale production development. A process based on siloxane polymerization using alternating exposures of tris(tert-pentoxy)silanol (TPS) and trimethylaluminum (TMA) enabled rapid SiO_2_ ALD with a significantly high GPC of 1.8 nm at 170 °C [15]. Accordingly, a SiO_2_ layer obtained for five cycles (i.e., ~9 nm) could entirely suppress the catalytic activity of TiO_2_ pigments. Compared to the conventional SiO_2_ ALD process [14], although a thicker film is needed to suppress the photocatalytic activity of TiO_2_, which could be due to the lower mass density, the significantly lower deposition temperature and the remarkably higher GPC provide a fast and efficient deposition method that is suitable for upscaling. Meanwhile, the mitigation of photocatalytic activity of TiO_2_ by thin Al_2_O_3_ films deposited by ALD was also investigated. Hakim et al. demonstrated that an Al_2_O_3_ layer obtained for 50 ALD cycles at 170 °C (GPC of 0.2 nm) could suppress the high photocatalytic activity of P25 TiO_2_ [40]. It is worth noting that, for pigment application, the thickness of the coating layer is of crucial importance: the layer must be thick enough to efficiently suppress the photocatalytic activity of TiO_2_, but thin enough to conserve the gloss and brightness of TiO_2_. This requires an optimal thickness, which is normally in the range of a few nanometers [17]. Therefore, reducing the coating thickness while ensuring its photocatalytic suppression ability is highly desirable.

This work reports on the suppression of photocatalytic activity of various types of TiO_2_ powders (i.e., anatase, rutile, and P25 TiO_2_) by Al_2_O_3_ films deposited via an ALD-like process using TMA and H_2_O as precursors, which was carried out at room temperature and atmospheric pressure in a home-built fluidized bed reactor. This gas-phase deposition process enabled the control of coating thickness at the sub-nanometer level, allowing us to investigate the dependence of photocatalytic suppression ability of Al_2_O_3_ on the film thickness. The surface morphology and film thickness, composition, and crystallinity of the coating layers were characterized by transmission electron microscopy (TEM), X-ray photoelectron spectroscopy (XPS) and thermogravimetric analysis (TGA), and X-ray diffraction spectroscopy (XRD), respectively. The results showed that highly conformal Al_2_O_3_ films with a thickness as thin as 1 nm were obtained, which efficiently suppressed the photocatalytic activity of TiO_2_ powders without affecting their bulk optical properties. Furthermore, the influence of high-temperature annealing on the properties of the Al_2_O_3_ layers was investigated, revealing the possibility to achieve porous Al_2_O_3_ layers, which could be useful for other applications.

## 2. Reaction Mechanism of Al_2_O_3_ ALD Using TMA and H_2_O: A Brief Overview

The high reactivity of TMA facilitates the deposition of Al_2_O_3_ in a broad range of temperature on various types of substrates and materials with any geometries, including flat surfaces [21], high-aspect-ratio structures [41,42,43], porous media [44,45,46,47], nanoparticles [29,32,34,40], fibers [48], carbon nanotubes [49,50], graphene [51,52,53], polymers [54], and biomaterials [55]. The reaction mechanism in ALD of Al_2_O_3_ using TMA and H_2_O has been intensively investigated in the past decades, both theoretically and experimentally [21,56,57,58,59,60,61,62,63,64,65,66,67]. Accordingly, the surface chemical reactions that lead to the deposition of Al_2_O_3_ in the ideal case can be divided into two half-reactions. During the exposure to TMA, the first half-reaction takes place and proceeds as:

║–OH + Al(CH_3_)_3_ (g) → ║–O–Al(CH_3_)_2_ + CH_4_ (g), (1)

where the ║–OH represents the functional groups (i.e., hydroxyl groups), which are formed during the exposure of the substrate to air, or surface pretreatment. After all of the –OH groups have reacted with TMA, the reactions achieve saturation, forming a –CH_3_ terminated surface, which is ready for the second half-reactions to take place during the exposure to H_2_O, described as:

║–O–Al–CH_3_ + H_2_O (g) → ║–O–Al–OH + CH_4_ (g). (2)

These reactions convert the –CH_3_ into CH_4_ and create a new surface terminated by –OH groups, which serves two purposes: blocking further reactions with H_2_O, which causes saturation, and providing an active surface for the chemical reactions in the next cycle. A typical GPC in the range of 0.1–0.3 nm is obtained for TMA/H_2_O ALD depending on experimental conditions such as temperature range, pressure range, and reactor type. The sequential exposures are repeated to deposit Al_2_O_3_ films, which translates into ability to control the film thickness at atomic level.

Nevertheless, the TMA/H_2_O ALD chemistry is rather complex, which involves—apart from the ligand-exchange reactions described above—dissociation reactions that can take place in both the half-reactions [21,68]. In the first half-reaction, TMA can react with unsaturated Al–O pairs of Al_2_O_3_ layers, creating a –CH_3_ terminated surface described as [68]:

║Al–O║ + Al(CH_3_)_3_ (g) → ║Al–CH_3_ + ║O–Al(CH_3_)_2_. (3)

In the second half-reaction, dissociation reactions can occur between H_2_O and the oxygen on Al_2_O_3_ surface, forming –OH surface functional groups, described as [21]:

║–O–║ + H_2_O (g) → 2║–OH. (4)

The reversed reaction of the dissociation, the dehydroxylation, can also take place that consequently reduces the concentration of the OH groups, especially at high temperatures [21]:

2║–OH → ║–O–║ + H_2_O (g). (5)

Although the dissociation reactions are not often mentioned in the literature when discussing the surface chemistry in TMA/H_2_O ALD, these reactions are believed to contribute considerably to growth. Especially, the dissociative chemisorption of TMA in the first half-cycle is the key mechanism that is used to interpret the nucleation on hydroxyl-free surfaces [59,60,69,70,71,72], even at room temperature [70]. In addition, at high deposition temperatures, the desorption of –OH groups and decomposition of TMA can occur simultaneously, which will strongly affect the growth rate of Al_2_O_3_ ALD [57,73]. Nevertheless, the ligand-exchange reactions are considered the dominant reactions during the deposition and have been most studied. 

The GPC in ALD regime is generally temperature-independent, which is commonly known as “*ALD window*” [22]. However, ALD of Al_2_O_3_ using TMA/H_2_O is found to be temperature-dependent [57,61,63,65]. Rahtu et al. [57] demonstrated a slight increase of GPC with increasing temperature from 150 to 250 °C, followed by a drop of GPC with further increase of temperature. The lower GPC at low temperature was attributed to the slow kinetics of the chemical reactions between the precursors, whereas the desorption of hydroxyl groups at high temperatures led to the drop of GPC. Most recently, the decrease of GPC at low deposition temperatures has been thoroughly investigated by Vandalon and Kessels [65]. Using in-situ vibrational sum-frequency generation technique, the authors investigated the reaction mechanism between TMA and H_2_O in a broad temperature range (i.e., 100–300 °C), which revealed that the low GPC is caused by the incomplete removal of –CH_3_ groups by H_2_O at low temperature. The unreacted –CH_3_ groups decline the chemisorption of TMA in the subsequent cycles, which consequently decreases the growth rate. The study also demonstrate that the persistent –CH_3_ groups are not accumulated with increasing the number of cycles, which explains the low carbon impurity in Al_2_O_3_ layers grown by ALD at low temperatures [65]. Nevertheless, the experimental data obtained by Ylivaara et al. have demonstrated that the low-temperature deposition of Al_2_O_3_ using TMA and H_2_O is inherently associated with a considerable amount of impurities, especially hydrogen and carbon, which increase with decreasing deposition temperature [74]. The hydrogen impurity arises from the unreacted hydroxyl groups, which has recently been verified by Guerra-Nunez et al. [66]. The degree of the GPC decrease at low temperatures is not well determined, and strongly depends on experimental conditions. For instance, contrary to the considerable drop of GPC at low temperatures observed by Vandalon and Kessels (i.e., the growth rate drops by approximately 50% when reducing the temperature from 250 to 100 °C), Groner et al. previously showed a slight decrease of GPC with decreasing temperature, even to near room temperature (i.e., 33 °C) [61]. The discrepancy between the studies can be attributed to the different ALD conditions, especially the process pressure range and reactor types.

## 3. Results and Discussion

### 3.1. Properties of the Al_2_O_3_ Coating Layers

#### 3.1.1. Morphology of Al_2_O_3_-Coated TiO_2_

**Figure 1:**
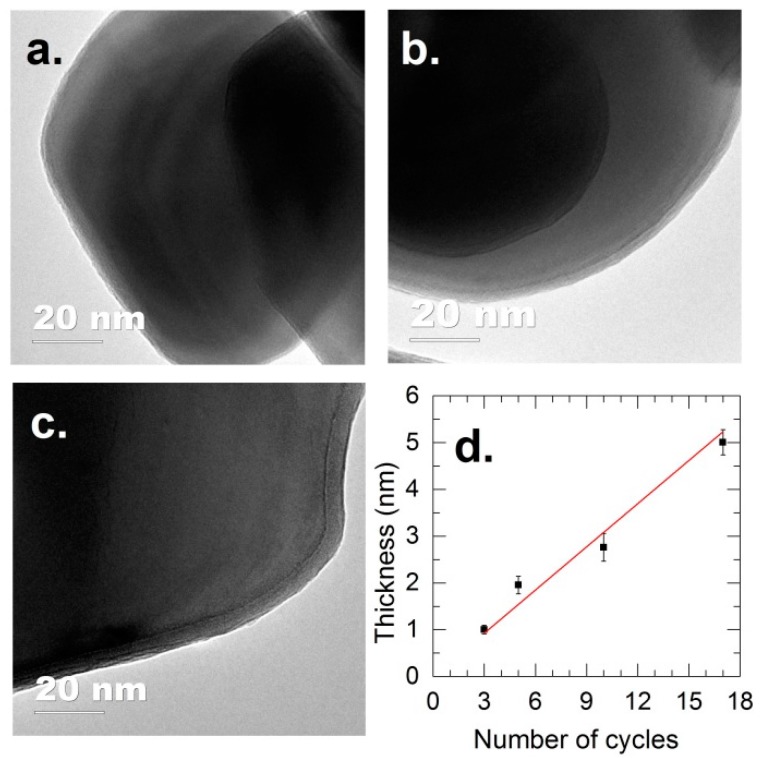
TEM images of Al_2_O_3_ films deposited on anatase TiO_2_ nanoparticles for (**a**) three cycles, (**b**) 10 cycles and (**c**) 17 cycles, and (**d**) the coating thickness as a function of the number of cycles.

We will first focus on the coating and performance of anatase TiO_2_ powders. The morphology of the Al_2_O_3_ films on anatase TiO_2_ for different exposure cycles is shown in Figure 1, demonstrating the deposition of uniform Al_2_O_3_ films, even at a film thickness of about 1 nm (Figure 1a). The coating thickness increases linearly with the number of cycles, with a GPC of approximately 0.3 nm (Figure 1d). The obtained GPC is higher than the GPC reported for ALD of Al_2_O_3_ on flat substrates, which is typically in the range of 0.1–0.2 nm [61,65]. However, this GPC value is lower than the GPC obtained for the Al_2_O_3_ ALD on particles (i.e., 0.5 nm) reported by Liang et al. [75]. Nevertheless, previous work from our group demonstrated that the growth of Al_2_O_3_ at room temperature and atmospheric pressure in FBR follows the chemical vapor deposition (CVD) mode, in which the GPC increases with increasing dosing time (i.e., without a self-saturating regime) [34]. With a GPC of 0.3 nm obtained for the examined conditions, a controlled deposition at sub-nanometer level of highly conformal Al_2_O_3_ films is totally achievable. In fact, this higher GPC is beneficial for the fast and scalable production that is required for practical applications.

#### 3.1.2. Structural and Optical Properties of Room-Temperature-Grown Al_2_O_3_

**Figure 2:**
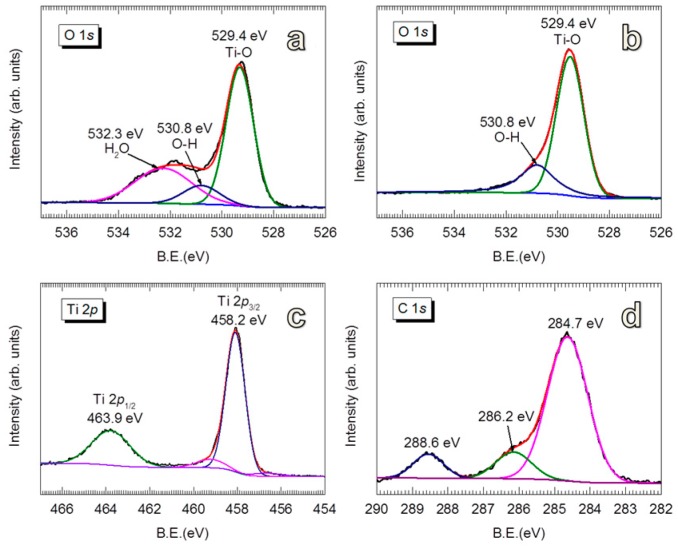
XPS spectra of uncoated anatase TiO_2_ powders: O 1*s* (**a**) without and (**b**) with preheating (at 170 °C in air), (**c**) Ti 2*p* and (**d**) C 1*s*.

The chemical states of the initial TiO_2_ surface are characterized by XPS and shown in Figure 2. The fingerprints of TiO_2_ are featured by the peaks at binding energies (BE) of 529.4 eV (O 1*s*, Figure 2a), BE = 463.9 eV and BE = 458.2 eV (Ti 2*p*, Figure 2c). The deconvolution of O 1*s* spectrum revealed the presence of a considerable amount of –OH groups (BE = 530.8 eV) and physisorbed H_2_O (BE = 532.3 eV) [76,77]. The physisorbed H_2_O can be removed by applying a heat treatment in air at 170 °C for 1 h, as indicated by Figure 2b. We note that no distinguishable difference was observed for the growth of the Al_2_O_3_ on the TiO_2_ without and with heat treatment. However, the existence of –OH groups on the surface is important to the inception of TMA chemisorption. The C 1*s* spectrum exhibits different states of C contamination (Figure 2d), including C=O (BE = 288.6 eV), O–C (BE = 286.2 eV) and C=C (BE = 284.7 eV) [76,78]. These carbon compounds could arise from adsorbed species on the surface of the powders and/or the carbon tape used to immobilize the TiO_2_ particles.

**Figure 3:**
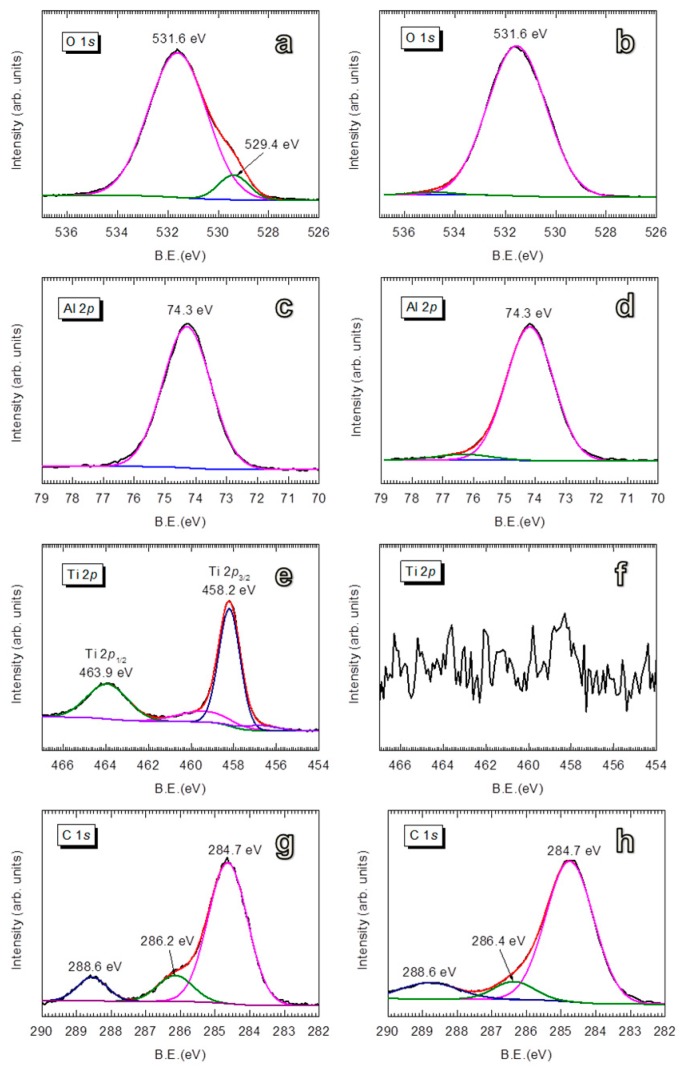
XPS spectra of anatase TiO_2_ coated with 3 nm thick (**a**,**c**,**e**,**g**—on the left side) and 5 nm thick (**b**,**d**,**f**,**h**—on the right side) Al_2_O_3_ films.

For the Al_2_O_3_-coated TiO_2_, the BE = 531.6 eV (for O 1*s*, Figure 3a,b) and BE = 74.3 eV (for Al 2*p*, Figure 3c,d) represent the Al–O bonds in Al_2_O_3_ compounds [78,79,80]. No noticeable change was observed for the BE of Al 2*p* with increasing coating thickness. However, the peaks of TiO_2_ (i.e., BE = 529.4 eV for O 1*s*, BE = 463.9 eV for Ti 2*p*_1/2_, and BE = 458.2 eV for Ti 2*p*_3/2_) are gradually attenuated with increasing Al_2_O_3_ thickness. For the TiO_2_ coated with 5 nm Al_2_O_3_, the TiO_2_ peaks are almost vanished, which suggests that the photoelectrons of TiO_2_ excited by the X-rays are effectively shielded by the Al_2_O_3_ film. The disappearance of TiO_2_ features in XPS spectra is evidence of uniform Al_2_O_3_ coating on a large scale, in addition to the evidence provided by the TEM micrographs shown in Figure 1. Furthermore, the gradual attenuation of C 1*s* at BE = 288.6 eV (C=O) and BE = 286.2 eV (O–C) relatively compared to the peak at BE = 284.7 eV (C=C) with increasing Al_2_O_3_ thickness can be explained that these carbon-containing species are on the surface of TiO_2_. This, however, does not rule out the possibility that the carbon content is accumulating in the growing film, which cannot be avoided due to the deposition at room temperature. 

**Figure 4:**
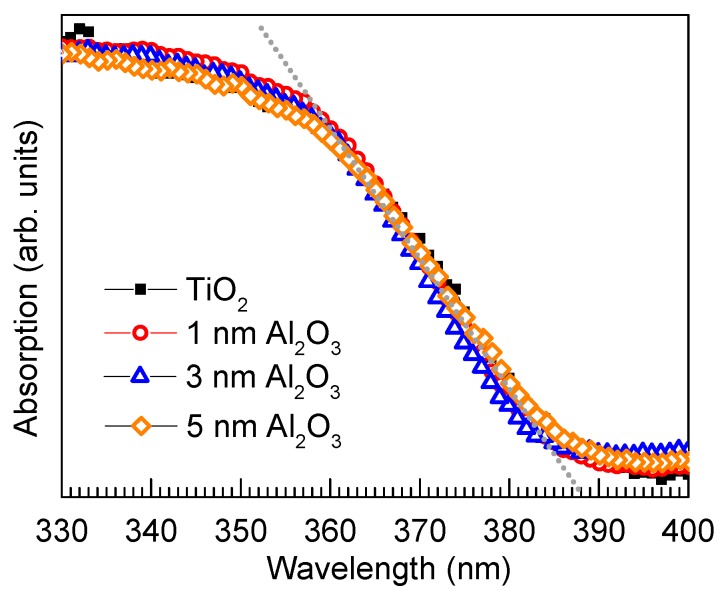
Absorption spectra of pristine anatase TiO_2_ powder and the TiO_2_ coated with Al_2_O_3_ films with different thicknesses: 1 nm, 3 nm, and 5 nm.

As a photocatalytic suppression layer for pigment applications, the transparency of the coating layer, which is crucially important to maintain the bulk optical properties of TiO_2_ such as the white color and high brightness, is highly desirable. The optical absorption spectra shown in Figure 4 indicate that the absorption of TiO_2_ remained unaffected upon the coating with Al_2_O_3_ with different film thicknesses. From the absorption spectra, an optical bandgap of 3.2 eV was determined, which corresponds to that of anatase TiO_2_ [81]. The characterization using X-ray diffraction showed the amorphous state of the Al_2_O_3_ layers, even after annealing at 500 °C for 12 h (Figure S1). 

#### 3.1.3. Thermogravimetric Analysis

**Figure 5:**
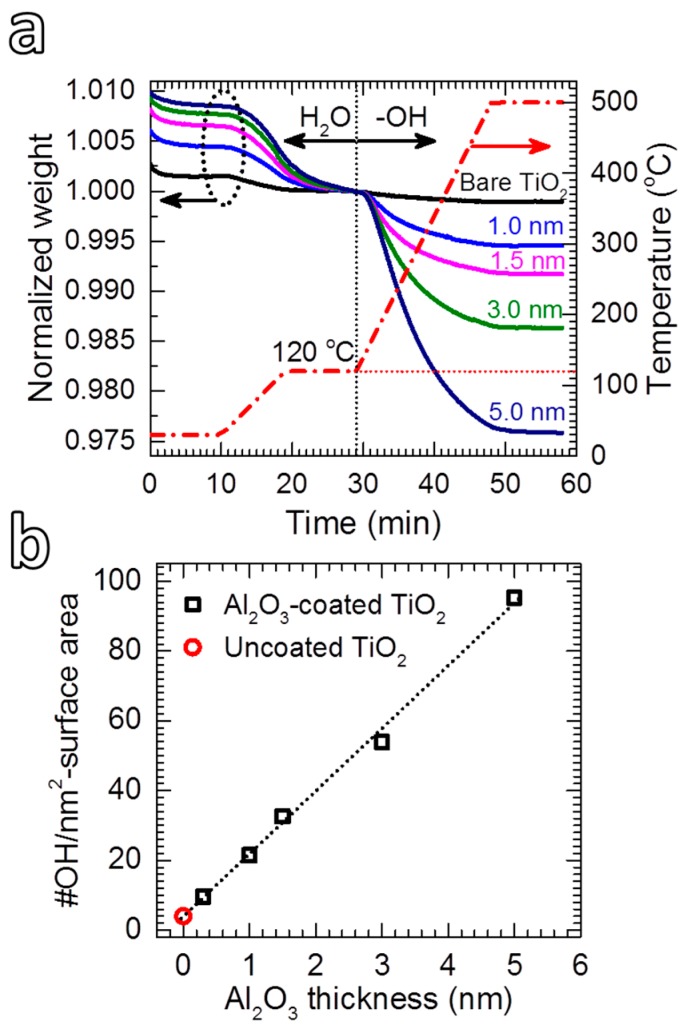
(**a**) TGA curves of uncoated and Al_2_O_3_-coated anatase TiO_2_ powders and (**b**) the density of hydroxyl groups as a function of Al_2_O_3_ thickness.

Figure 5a shows TGA plots of the TiO_2_ powders coated with Al_2_O_3_ films with different thicknesses. The first stage (i.e., T ≤ 120 °C) describes the desorption of water when the powders are heated from 25 °C, showing an increasing amount of water with Al_2_O_3_ thickness. The second stage demonstrates the desorption of hydroxyl groups, which increases remarkably with increasing the coating thickness. Following the calculation method proposed by Pratsinis et al., from the TGA plots shown in Figure 5a, the density of –OH groups was estimated by following the common assumption that all the –OH groups are on the surface [82,83]. For uncoated TiO_2_, a density of 4 –OH/nm^2^ was obtained, which is close to the value reported for anatase TiO_2_ [83]. On Al_2_O_3_-coated TiO_2_, the density of –OH groups increased linearly with Al_2_O_3_ thickness (Figure 5b): a density of 9.6 –OH/nm^2^ was obtained for the 1 nm thick Al_2_O_3_ coated powder, and increased to 95.3 –OH/nm^2^ for the TiO_2_ coated with 5 nm Al_2_O_3_. As this number is not reasonable for the assumption that all the –OH groups are on the surface of the powder (i.e., it is theoretically and practically impossible to have 95 OH –groups on 1 nm^2^), the calculated values suggest that most of the –OH groups are located inside the coating layer. This is caused by the incomplete consumption of –OH groups, which leads to hydrogen impurity in the film [66]. The existence of –OH strongly affects the density of Al_2_O_3_ [61]. Nevertheless, Groner et al. [61] demonstrated that the Al_2_O_3_ films grown at low temperatures exhibited excellent electrical properties despite containing high –OH concentrations. As we will demonstrate later, despite having high densities of –OH groups, ultrathin Al_2_O_3_ films deposited at room temperature can provide excellent photocatalytic suppression ability.

### 3.2. Photocatalytic Activity of Al_2_O_3_-Coated TiO_2_

#### 3.2.1. Dependence of Photocatalytic Suppression Ability of Al_2_O_3_ on Film Thickness

**Figure 6:**
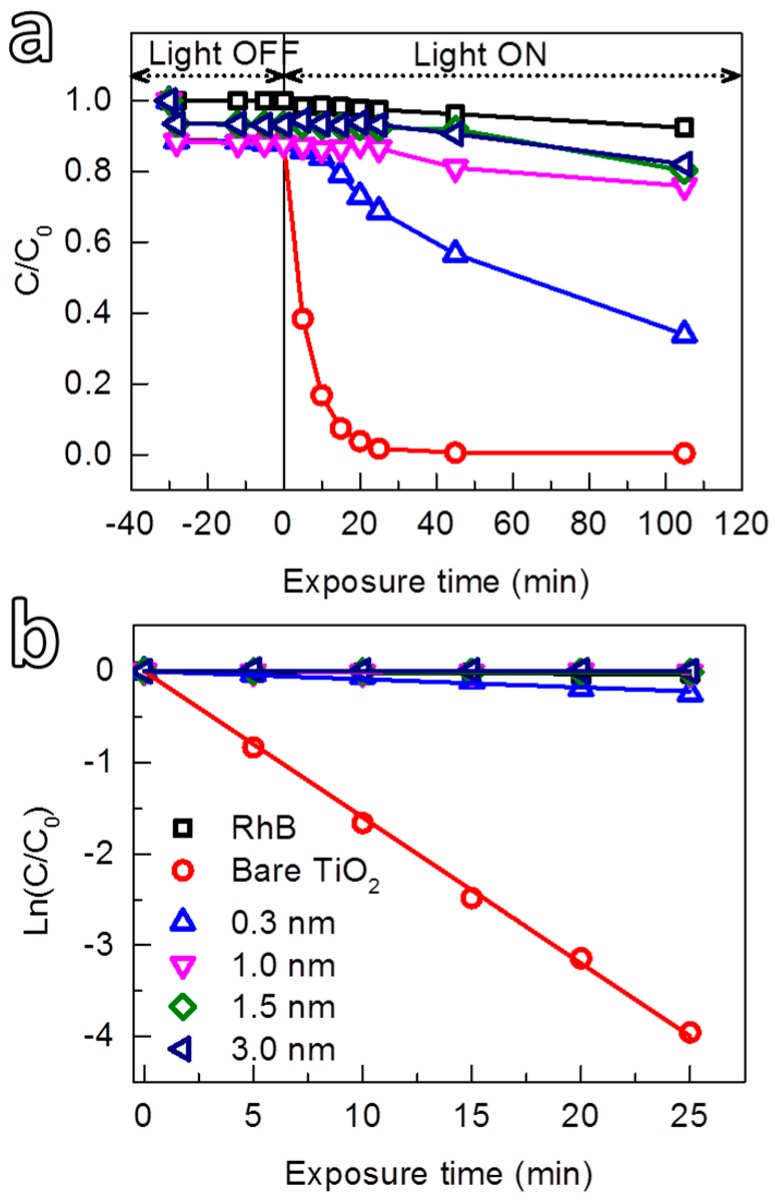
(**a**) Photodegradation of RhB solution using anatase TiO_2_ coated with Al_2_O_3_ films with different thicknesses as a function of exposure time, and (**b**) the corresponding kinetic reaction plots.

The photocatalytic suppression ability of the room-temperature grown Al_2_O_3_ was investigated by the photodegradation of rhodamine B (RhB) solution under the irradiation of sunlight generated by a solar simulator. The ability to control the growth at sub-nanometer level allowed us to study the interdependence of the photocatalytic suppression ability and the thickness of Al_2_O_3_. Figure 6 shows the photocatalytic activity toward the photodegradation of RhB of TiO_2_ powders coated with Al_2_O_3_ films with different thicknesses. For comparison, the self-degradation of RhB (i.e., without TiO_2_) caused by the UV light and the photocatalytic activity of uncoated TiO_2_ were also investigated. Prior to the UV irradiation, the solution was continuously stirred in the dark (i.e., light-off stage) for 30 min to obtain adsorption/desorption equilibrium of RhB and uniform suspensions, which were collected after certain time-intervals to determine the concentration of the residual RhB. The results showed that during the light-off stage, the concentration of RhB in the solution without TiO_2_ powders (blank RhB) remained unchanged. However, a small drop of RhB concentration was observed for the solutions with TiO_2_ powders (both uncoated and coated with Al_2_O_3_). This drop is due to the adsorption of a fraction of RhB molecules on the surface of the particles. Shortly after that (i.e., after 2 min) the concentration of RhB reached the steady state. In the light-on stage, a rapid decomposition of RhB in the solution containing uncoated TiO_2_ was observed (the circles in Figure 6a), and after 25 min of irradiation, the RhB was completely decomposed, indicative of the high photocatalytic activity of the TiO_2_. 

For the Al_2_O_3_-coated TiO_2_, the photocatalytic activity was reduced, which exhibited a strong dependence on the thickness of the Al_2_O_3_ layer. This thickness dependence can be quantitatively estimated by the kinetics of the photodegradation reaction, which can be described by first-order kinetics [84]:

ln(*C*_0_/*C*) = *k*_app_·*t*, or *C* = *C*_0_·exp(−*k*_app_·*t*), (6)

where *C*_0_ and *C* are the initial concentration and the concentration at the time *t*, respectively, *k*_app_ is the apparent first-order kinetic constant that represents the reaction rate. The kinetic plots describing the degradation of RhB are shown in Figure 6b, from which the *k*_app_ value for each reaction was extracted and presented in Table 1. Accordingly, the photocatalytic activity of TiO_2_ (*k*_app_ ≈ 160 × 10^−3^ min^−1^) was reduced approximately 18 times (to *k*_app_ ≈ 8.87 × 10^−3^ min^−1^) by coating an Al_2_O_3_ layer for 1 cycle of TMA and H_2_O exposures (i.e., the calculated thickness of about 0.3 nm). Nevertheless, the Al_2_O_3_ at this thickness cannot entirely suppress the photocatalytic activity of TiO_2_. This could be due to (1) the insufficient thickness that can block the transport of photogenerated charges from the TiO_2_ to the surface, and/or (2) the lack of continuity of the coating layer. With increasing film thickness, *k*_app_ value decreased rapidly, which dropped to far below 1 × 10^−3^ min^−1^ for Al_2_O_3_ films with a thickness of 1 nm or thicker (thickness *a* ≥ 1 nm). The results showed that an Al_2_O_3_ layer with a thickness of 1 nm efficiently suppressed entirely the catalytic activity of the TiO_2_. The degradation of RhB observed for the TiO_2_ coated with Al_2_O_3_ films with thickness *a* ≥ 1 nm is identical to that of the self-degradation of RhB solution without the powder (Figure 6b and Table 1). A comparison with other coating processes and materials that have been used to mitigate the photocatalytic activity of TiO_2_ pigments is given in Table 2. It is can be seen that gas-phase deposition methods such as ALD and CVD have been employed. However, the reported processes were carried out at much higher temperatures, i.e., above 150 °C for Al_2_O_3_ and 175 °C for SiO_2_. In addition, thicker layers are needed to efficiently suppress the photocatalytic activity of TiO_2_, which is also the case for the layer obtained by wet-chemistry methods. This indicates that our approach has significant advantages in providing a fast and efficient coating method that can be carried out at room temperature, which increases the feasibility for further development to large-scale production.

#### 3.2.2. Influence of High-Temperature Calcination on Photocatalytic Suppression Ability of Al_2_O_3_

As demonstrated by the thermal analysis shown in Figure 5, the room-temperature-grown Al_2_O_3_ films contain a high density of –OH groups, which can desorb at high temperatures. Therefore, a calcination at 500 °C for 12 h was applied to investigate the influence of the –OH desorption on the suppression ability of Al_2_O_3_. The results show that the calcination of Al_2_O_3_-coated TiO_2_ resulted in an enhanced photocatalytic activity (Figure S2), in which *k*_app_ values increased nearly an order of magnitude (Table 1). This indicates that the –OH desorption reduced the suppression ability of the Al_2_O_3_ layer. We speculate that the desorption of –OH groups resulted in the formation of porous Al_2_O_3_ that allows photogenerated electrons to transport to the surface. In addition, the densification, which my introduce cracks during calcination, can also take place and increase the porosity of the Al_2_O_3_ [74]. This is supported by the XPS spectra of the calcined Al_2_O_3_/TiO_2_ shown in Figure S3. Accordingly, the two XPS peaks of the Ti 2*p* core-levels that were attenuated after coating with a 5-nm Al_2_O_3_ film were partially recovered after the calcination, which suggests that the photoelectrons generated by the X-rays could travel through the calcined Al_2_O_3_ layer. For uncoated TiO_2_, the results show that the –OH desorption reduced the photocatalytic activity (Figure S2), indicated by the drop of *k*_app_ from 160 × 10^−3^ to 100 × 10^−3^ min^−1^ (Table 1). This can be explained by the fact that in TiO_2_ photocatalysis, hydroxyl groups act as hole traps, which causes two important effects that enhance the photocatalytic activity: enhancement of charge separation and formation of hydroxyl radicals [76,88,89,90].

#### 3.2.3. Photocatalytic Suppression Ability of Al_2_O_3_ on P25 and Rutile TiO_2_

**Figure 7:**
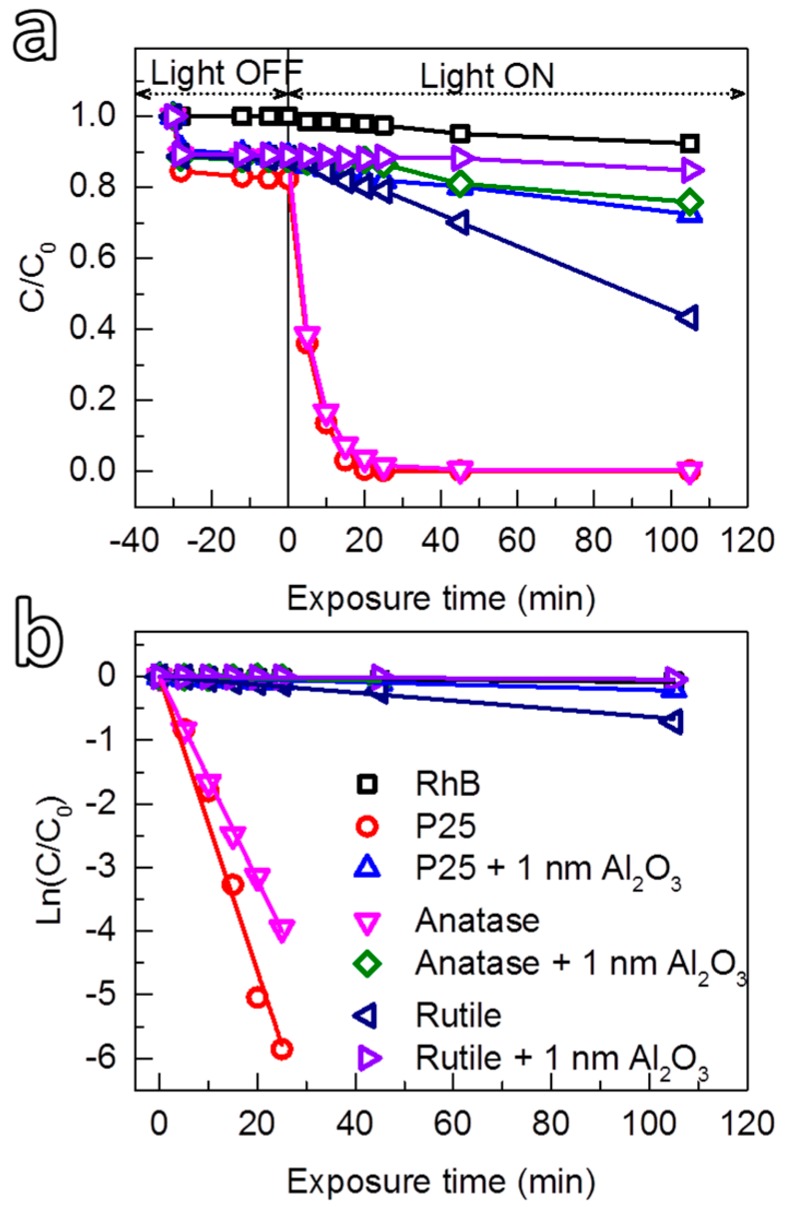
(**a**) Photodegradation as a function of exposure time of RhB solution by uncoated TiO_2_ (P25, anatase and rutile) and the TiO_2_ coated with 1 nm Al_2_O_3_, and (**b**) the corresponding kinetic plots. All of the photocatalytic tests were carried out under identical experimental conditions. The self-degradation of RhB is added as the reference.

Analogously to the deposition on anatase TiO_2_, uniform Al_2_O_3_ films were also achieved on rutile and P25 TiO_2_ powders (Figures S4 and S5), which enabled the study on photocatalytic suppression ability of the Al_2_O_3_ films on P25 and rutile TiO_2_ nanoparticles. The results are demonstrated in Figure 7. The photocatalytic activity of rutile TiO_2_ (*k*_app_ ≈ 4.85 × 10^−3^ min^−1^) is much lower than that of anatase TiO_2_ (*k*_app_ ≈ 159.88 × 10^−3^ min^−1^) under identical conditions, which is also known from the literature [90,91]. After coating the rutile particles with 1 nm Al_2_O_3_, the photocatalytic activity is strongly suppressed, which is confirmed by the low *k*_app_ value obtained (0.28 × 10^−3^ min^−1^). In contrast, the photocatalytic activity of P25 TiO_2_ (*k*_app_ ≈ 231 × 10^−3^ min^−1^) was found to be higher than that of anatase TiO_2_. Nevertheless, by coating with an Al_2_O_3_ layer with a thickness of approximately 1 nm, the photocatalytic activity of P25 was also strongly suppressed (*k*_app_ ≈ 2.01 × 10^−3^ min^−1^). These results have further demonstrated the high photocatalytic suppression ability of the ultrathin Al_2_O_3_ films deposited under mild conditions.

## 4. Experimental Section

The deposition was carried out in a home-built fluidized bed reactor operating at atmospheric pressure and room temperature, as described elsewhere [34]. Briefly, the system consisted of a glass column (26 mm in internal diameter and 500 mm in height) placed on top of a single motor Paja PTL 40/40–24 vertical vibration table to assist the fluidization of nanoparticles. Anatase (mean diameter *d* ≈ 270 nm) and rutile (mean diameter *d* ≈ 300 nm) TiO_2_ pigment powders were provided by Taihai TiO_2_ Pigment Co. (Panzhihua, China); aeroxide P25 TiO_2_ (mean diameter *d* ≈ 21 nm) were purchased from Evonik Industries (Hanau, Germany). Semiconductor grade trimethylaluminum (TMA) was provided by Akzo Nobel HPMO (Amersfoort, The Netherlands) in a 600 mL WW-600 stainless steel bubbler. Both the TiO_2_ powders and the TMA precursor were used as received. Pressurized nitrogen (99.999 vol %) was used as the carrier gas. For each experiment on anatase and rutile TiO_2_, 7.0 g of powders was used. An optimized N_2_ gas flow of 0.5 L min^−1^ was introduced through the distributor plate placed at the bottom of the glass column to fluidize the powders. A coating cycle consisted of alternating exposures of the powders to TMA precursor (2 min) and deionized water vapor (2 min), separated by a purging step (5 min) using N_2_. The coating on P25 followed the conditions described elsewhere [34]. The temperature inside the bed was monitored by a type-K thermocouple inserted in the column, which showed a small variation during the deposition (i.e., 27 ± 3 °C), possibly due to the heat release from the chemical reactions.

As-coated Al_2_O_3_/TiO_2_ powders were suspended in ethanol and transferred to regular transmission electron microscopy (TEM) grids (3.05 mm in diameter). TEM images were taken at several locations on the grids using a JEOL JEM1400 transmission electron microscope (JEOL, Peabody, MA, USA) operating at a voltage of 120 kV and a current density of 50 pA cm^−2^.

X-ray photoelectron (XPS) characterizations were carried out using a ThermoFisher K-Alpha system (ThermoFisher Scientific, Waltham, MA, USA) using Al Kα radiation with photon energy of 1486.7 eV. A sufficient amount of powder was immobilized on carbon tape before loading into the XPS chamber. Survey scans were acquired using a 200 μm spot size, 55 eV pass energy and 0.1 eV/step with charge neutralization. The peaks positions were calibrated according to the C 1*s* peak at 284.7 eV. 

A Mettler Toledo TGA/SDTA 851e thermogravimetric analyzer (Mettler Toledo B.V., Tiel, The Netherlands) was used for studying the thermal behavior of the synthesized powders. An amount of 30 mg of powders was used for each TGA measurement. The TGA curves were recorded while (1) heating up the powders from 25 to T_1_ = 120 °C with a ramping rate of 10 °C min^−1^ in a N_2_ flow of 100 mL min^−1^; (2) maintaining at 120 °C for 10 min; (3) ramping up to 500 °C with a ramping rate of 20 °C min^−1^; and (4) finally maintaining at this temperature for 10 min. Steps (1) and (3) are commonly used to determine the density of physisorbed water and chemisorbed hydroxyl groups on the surface, respectively [83].

The Al_2_O_3_/TiO_2_ was transferred onto a Si wafer with 300 nm of SiO_2_ thermal oxide, which was to eliminate the influence of the substrate (Si) signal in the X-ray diffractograms of the powders. The X-ray diffractograms were obtained by a PANalytical X’pert Pro diffractometer (PANalytical, Almelo, The Netherlands) with Cu Kα radiation, secondary flat crystal monochromator and X’celerator RTMS Detector system. The angle of interest 2θ was measured from 10° to 90° with fine steps of 0.001°.

Optical absorption measurements were performed using a PerkinElmer-Lambda 900 spectrometer (PerkinElmer, Spokane, WA, USA) equipped with an integrated sphere device. The powder was suspended in ethanol, which was transferred onto a quartz substrate and dried in air. The spectra were acquired in the wavelength range of 600–200 nm, with fine steps of 1 nm.

The photocatalytic activity of the Al_2_O_3_-coated TiO_2_ powders was evaluated by the photodegradation of RhB solution. For each test, 30 mg of the powders were added to 30 mL RhB solution (concentration of 8 mg L^−1^) and continuously stirred in the dark for 30 min to obtain a uniform suspension. Thereafter, the suspension was exposed to UV radiation generated by a mercury lamp with a power of 45 W for different exposure times. The set-up allowed us to assess up to 10 samples simultaneously, which ensured that all samples were irradiated under the same conditions, such as light intensity, exposure time, and temperature. The suspension was then centrifuged to separate the powders from the solution. Finally, the solution was analyzed by UV-visible spectrophotometry (HACH LANGE DR5000 UV-vis spectrometer, Hach-Lange GmbH, Düsseldorf, Germany) to determine the residual concentration of the RhB in solution, which was used to evaluate the catalytic activity suppression of the Al_2_O_3_ layers.

## 5. Conclusions

We have demonstrated the deposition and investigated the photocatalytic suppression ability of ultrathin Al_2_O_3_ films on different types of TiO_2_ powders: anatase (mean diameter *d* ≈ 270 nm), rutile (mean diameter *d* ≈ 300 nm) and P25 (mean diameter *d* ≈ 21 nm). The deposition was carried out using an ALD-like process, in which the TiO_2_ powders were alternatively exposed to TMA and H_2_O at room temperature and atmospheric pressure in a FBR. This enabled the deposition of ultrathin, conformal and uniform Al_2_O_3_ films with the thickness control at sub-nanometer level. The deposition at room temperature resulted in amorphous Al_2_O_3_ layers containing a high concentration of hydroxyl groups, which was caused by the incomplete reactions between the precursors at low temperature. Nevertheless, the films exhibited excellent photocatalytic suppression ability, which showed that an Al_2_O_3_ layer with a thickness of 1 nm could efficiently suppress the photocatalytic activities of the TiO_2_ powders. In addition, the optical absorption of TiO_2_ was not affected by the Al_2_O_3_ coating. Upon calcination at a high temperature, photocatalytic suppression ability of the Al_2_O_3_ was decreased, possibly due to the formation of porous Al_2_O_3_ layer, which created pathways for charge carriers to transport to the surface. Our approach of using a FBR operating at atmospheric pressure is a fast, efficient, simple method for depositing ultrathin conformal Al_2_O_3_ films that meet the requirements for coating pigments, which can be further developed for large-scale production.

## Figures and Tables

**Table 1:** Apparent first-order rate constant, *k*_app_, of anatase TiO_2_ powders coated with Al_2_O_3_ with different film thicknesses.

Sample	*k*_app_ × 10^3^/min^−1^		*R*^2^ of Fitting	
	As-Deposited	Calcined	As-Deposited	Calcined
RhB	0.52 ± 0.03	0.52 ± 0.03	0.99	0.99
Uncoated TiO_2_	160 ± 1.60	100 ± 4.12	1.00	0.99
0.3 nm Al_2_O_3_ coated TiO_2_	8.87 ± 0.71	19.3 ± 0.03	0.96	0.99
1.0 nm Al_2_O_3_ coated TiO_2_	0.64 ± 0.21	7.07 ± 0.53	0.90	0.96
1.5 nm Al_2_O_3_ coated TiO_2_	0.41 ± 0.02	4.77 ± 0.68	0.99	0.89
3.0 nm Al_2_O_3_ coated TiO_2_	0.35 ± 0.03	3.27 ± 0.80	0.98	0.72

**Table 2:** Coating methods and materials for suppressing photocatalytic activity of TiO_2_ pigments.

TiO_2_ Material	Average Diameter (nm)	Coating Method	Coating Material	Coating Thickness (nm)	Deposition Temperature (°C)	Photocat. Reaction	Ref.
P25	21	ALD	Al_2_O_3_	6.0	177	Methylene blue	[40]
Anatase Rutile	160 280	ALD	SiO_2_	2.0	500 for SiO_2_ 177 for Al_2_O_3_	IPA to acetone	[14]
			SiO_2_/Al_2_O_3_	1.0/1.0			
			SiO_2_/Al_2_O_3_/SiO_2_/Al_2_O_3_	0.5/0.5/0.5/0.5			
P25	21	ALD	Al_2_O_3_	3.8	150	RhB	[85]
Anatase	160	ALD	SiO_2_	6.0	175	Methylene blue	[15]
P25	18			9.0			
Anatase	160	MLD	Alucone	7–10	100–160	Methylene blue	[86]
Rutile	Not reported	CVD	SiO_2_	1–2	900–1000	Methylene blue	[13]
Rutile	300	Wet-chemistry	ZrO_2_	5.0	40	RhB	[19]
			CeO_2_	1–2	60		
Rutile	300	Wet-chemistry	CeO_2_	1–2	60	RhB	[87]
ST-21	20	Wet-chemistry	SiO_2_	4.0	40	Methylene blue	[8]
P25	21	Wet-chemistry	Porous SiO_2_	20.0	Room temperature	RhB	[10]
Anatase Rutile P25	270 300 21	ALD-like	Al_2_O_3_	1.0	Room temperature	RhB	This work

## Supplementary Materials

The following are available online at http://www.mdpi.com/2079-4991/8/2/61/s1, Figure S1: XRD patterns of uncoated TiO_2_ and Al_2_O_3_-coated TiO_2_, with or without annealing at 500 °C for 12 h. Figure S2: The comparison of photocatalytic activity of Al_2_O_3_/TiO_2_ (anatase) powders before and after annealing at 500 °C for 12 h. Figure S3: XPS spectra of C 1*s*, Al 2*p*, O 1*s* and Ti 2*p* core-levels of anatase TiO_2_ coated with 5 nm Al_2_O_3_ as-deposited and after annealing at 500 °C for 12 h in air. Figure S4: TEM micrographs of rutile TiO_2_ coated with Al_2_O_3_ films deposited for 3 cycles, 5 cycles, 10 cycles and 17 cycles. Figure S5: High-resolution TEM micrographs of P25 TiO_2_ nanoparticles coated with Al_2_O_3_ for 4 cycles, 7 cycles and 15 cycles.
