# Supplementary material for: Suppressing the Photocatalytic Activity of TiO2 Nanoparticles by Extremely Thin Al2O3 Films Grown by Gas-Phase Deposition at Ambient Conditions
Source: Nanomaterials (Basel). 2018 Jan 24;8(2):61. doi: 10.3390/nano8020061 (PMC5853694; doi:10.3390/nano8020061)
Supplement: Supplementary file 1 [file nanomaterials-08-00061-s001.pdf]

Suppressing the Photocatalytic Activity of TiO_2 Nanoparticles by Extremely Thin Al_2O_3 Films Grown by Gas-Phase Deposition at Ambient Conditions

Jing Guo[1,2,4], Hao Van Bui[1,5*], David Valdesueiro[1,3], Shaojun Yuan[2], Bin Liang[2], and J. Ruud van Ommen[1]

[1] Product & Process Engineering, Department of Chemical Engineering, Faculty of Applied Sciences, Delft University of Technology, 2629 HZ Delft, The Netherlands
[2] Multi-phase Mass Transfer & Reaction Engineering Lab, College of Chemical Engineering, Sichuan University, 610065, Chengdu, China
[3] Delft IMP B.V., Molengraaffsingel 10, 2629 JD Delft, The Netherlands
[4] Shanxi Province Key Laboratory of Higee-Oriented Chemical Engineering, North University of China, Taiyuan, Shanxi 030051, China
[5] Department of Physics, Quy Nhon University, 170 An Duong Vuong Street 590000, Quy Nhon City, Vietnam

*Corresponding author: V.H.Bui@tudelft.nl

Supporting Information

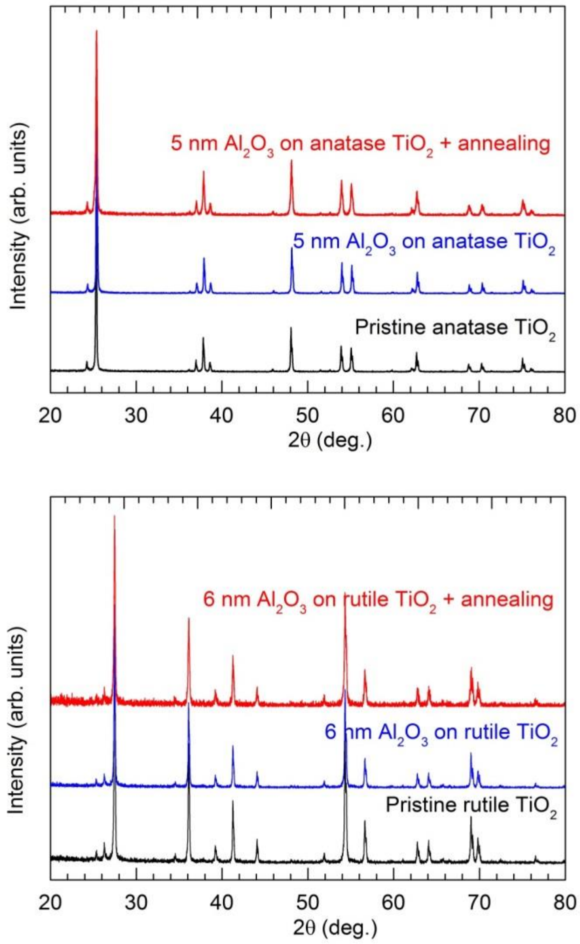

Figure S1. XRD patterns of uncoated TiO_2 and Al_2O_3-coated TiO_2, with or without annealing at 500 °C for 12 hours: anatase (top) and rutile (bottom). No signature of Al_2O_3 crystal phase was observed, suggesting the amorphous state of the coating layers. The coating has no effects on the crystalline structure of the TiO_2, even after the annealing.

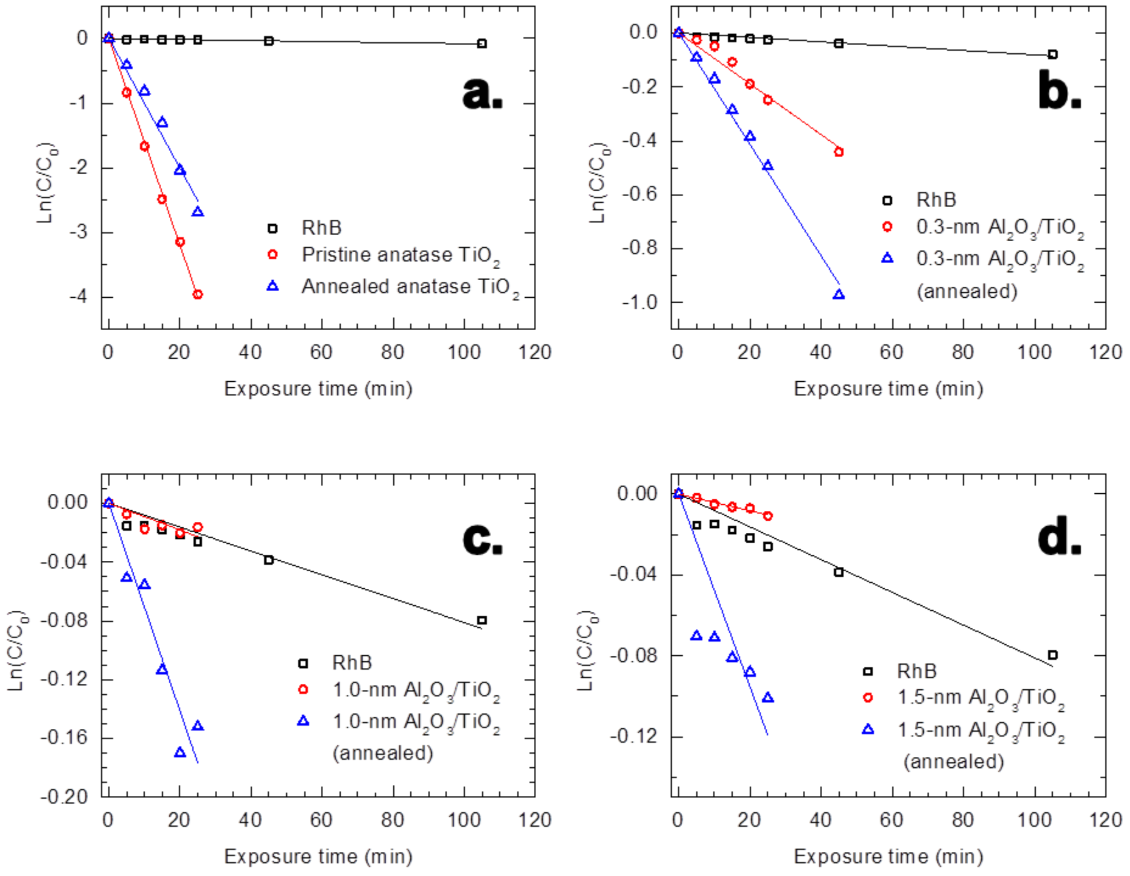

Figure S2. The comparison of photocatalytic activity of Al_2O_3/TiO_2 (anatase) powders before and after annealing at 500 °C for 12 hours: (a) uncoated anatase TiO_2; (b) TiO_2 coated with 0.3 nm Al_2O_3 (i.e., 1 ALD cycle, this value of thickness is estimated based on the growth-per-cycle (GPC) of 0.3 nm); (c) TiO_2 coated with 1 nm Al_2O_3; and (d) TiO_2 coated with 1.5 nm Al_2O_3. From these plots, it is better seen that the degradation of the TiO_2 coated with an Al_2O_3 layer with a thickness of about 1 nm is identical with the self-degradation of RhB. The lower slope obtained for 1.5-nm Al_2O_3 coated TiO_2 compared to blank RhB (Figure d) is due to the contribution of incomplete removal of the particles by centrifugation. In this case, the residual particles virtually act as an "extra amount of RhB" that detected by the UV-Vis measurement. This effect is only visible when the degradation caused by the photocatalyst is negligible.

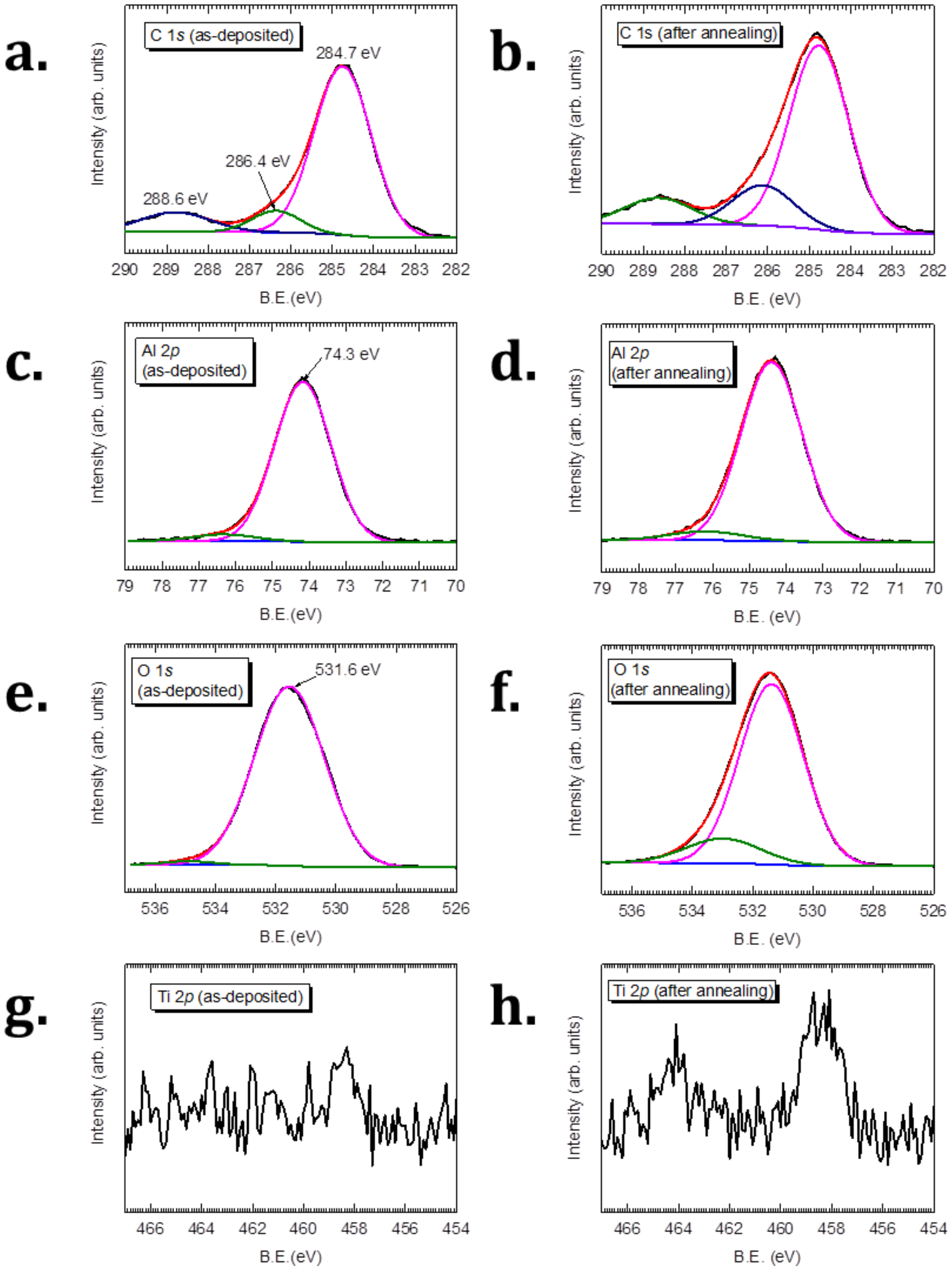

Figure S3. XPS spectra of C 1*s*, Al 2*p*, O 1*s* and Ti 2*p* core-levels of anatase TiO_2 coated with 5 nm Al_2O_3: as-deposited (a, c. e & g) and after annealing at 500 °C for 12 hours in air (b, d, f & h). No significant changes were observed for the spectra of C 1*s* and Al 2*p* after annealing.

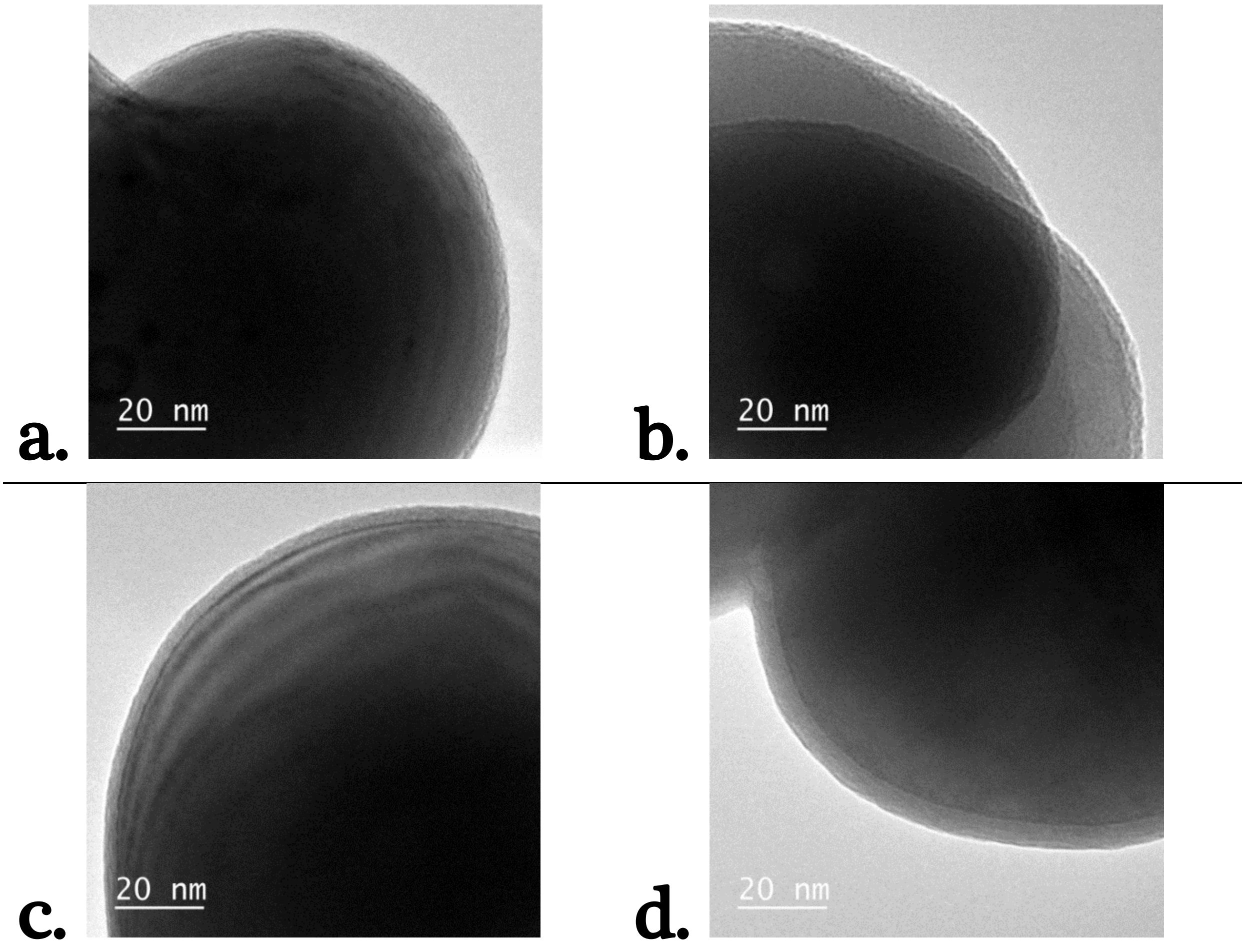

Figure S4. TEM micrographs of rutile TiO_2 coated with Al_2O_3 films deposited for (a) 3 cycles, (b) 5 cycles, (c) 10 cycles and (d) 17 cycles. The results demonstrate the growth of uniform Al_2O_3 films. A GPC of 0.34 nm was obtained for the deposition on rutile TiO_2 under the experimental conditions applied for the deposition on anatase TiO_2.

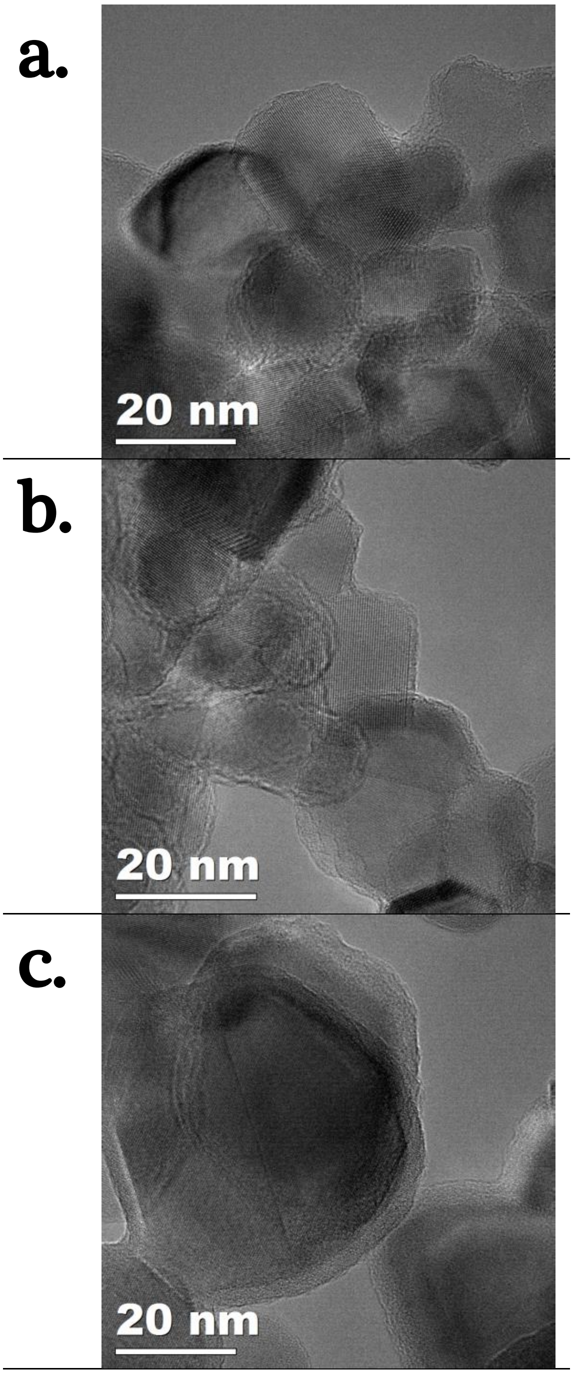

Figure S5. High-resolution TEM micrographs of P25 TiO_2 nanoparticles coated with Al_2O_3 for (a) 4 cycles, (b) 7 cycles and (c) 15 cycles. The growth mechanism on P25 TiO_2 was reported elsewhere.[1] We observed that, in comparison with the growth on anatase and rutile TiO_2, the uniformity of Al_2O_3 films on P25 TiO_2 nanoparticles is lower. Nevertheless, an Al_2O_3 layer with a thickness of approximately 1 nm obtained for 4 cycles could sufficiently suppress the high photocatalytic activity of the P25.

References

(1) Valdesueiro et al., Gas-Phase Deposition of Ultrathin Aluminium Oxide Films on Nanoparticles at Ambient Conditions. *Materials* **2015**, *8*, 1249-1263.
